# Editorial: Defects in Regulation: How, Where and When the Immune System Can Go Wrong

**DOI:** 10.3389/fimmu.2021.746418

**Published:** 2021-08-13

**Authors:** Ger T. Rijkers, Carlo Riccardi, Frans G. M. Kroese

**Affiliations:** ^1^Department of Science, University College Roosevelt, Middelburg, The Netherlands and Microvida Laboratory for Medical Microbiology and Immunology, St. Elisabeth Hospital, Tilburg, Netherlands; ^2^Department of Medicine, University of Perugia, Perugia, Italy; ^3^Department of Rheumatology and Clinical Immunology, University of Groningen, University Medical Center Groningen, Groningen, Netherlands

**Keywords:** immunoregulation, autoinflammation, autoimmunity, AIRE, glucocorticoids, TNF-blockade, addison, SLE

The immune system is a highly complex cellular and molecular machinery conferring protection against infectious diseases and tumors. Like other complex machineries, the immune system requires strict regulation and guidance to function properly and to prevent it from going wrong. How, where and when can the immune system go wrong?

Crucial in keeping the level of immune- and inflammatory responses within the physiological range is the balance between different kinds of myeloid and lymphoid regulatory cells, including T regulatory cells, and antigen presenting cells. Moreover other cells, including for example dendritic cells, macrophages and neutrophils, also can contribute to the immune/inflammatory response and diseases.

Examples of where and when the immune system can go wrong are manifold. Defective expression of the autoimmune regulator (AIRE) gene, which prevents thymic transcription of tissue-specific self-antigens, disables the proper sensing of “self” and results in autoreactivity and self-destruction. Uncontrolled proliferation of immune cells may result in immunoproliferative diseases, such as leukemia. A compromised immune responses lead to immunodeficiencies including the autoinflammatory periodic fever syndromes. Finally, overactivation of the immune system may result in autoimmune diseases or allergies.

The aim of this Research Topic was to provide further insight into the relation between physiological functions of the immune system and the failure of regulatory mechanisms leading to immunodeficiency and autoimmunity with a focus on autoinflammation.

Discovery of a genetic defect in genes involved in immune responses may (as an “experiment of nature”) lead to a better understanding of the physiology of the immune system. Thus, a defect in AIRE could explain how selection against recognition of self could be realized in the thymus. In addition, defects may also help to unravel the pathophysiology of immune mediated diseases. Thus, AIRE mutations link molecular defects to the pathophysiology of polyglandular autoimmune diseases such as Addison’s disease. Perniola et al. review the history and current evidence of the role of AIRE in autoimmune Addison’s disease as Part of the Autoimmune Polyglandular Syndrome Type 1.

In other cases, a given genetic defect may not point directly to the pathophysiological mechanism of the disease. Martirosyan et al. from Yerevan in Armenia have studied the transmigration of neutrophils in patients with Familial Mediterranean Fever (FMF). FMF is caused by mutations in the pyrin encoding MEFV gene. Their data show that the mutated pyrin inflammasome is highly sensitive for slight changes in the cytoskeleton, even in the absence of pathogens. This could explain the apparent autoinflammatory nature of this disease.

Overexpression of interferon, based on gain-of-function mutations in *STING1* (Stimulator of interferon response cGAMP interactor), causes a rare autoinflammatory interferonopathy with systemic inflammation, vasculopathy and interstitial lung disease. Lin et al. from NIH in Bethesda with an international team of researchers now have found novel variants at several domains of the molecule, including the transmembrane domain, which also lead to STING autoactivation and vascular disease in the absence of ligand binding.

Epigenetic modifications also can be associated with autoimmune conditions. Kakan et al. have investigated miRNA signatures in the NOD mouse model of Sjögren’s syndrome and suggest that upregulation of a set of five miRNAs dysregulates inflammation pathways, likely contributing to systemic inflammation and lymphocytic infiltration of the exocrine glands. Yu et al. from Jinan University in Shenzhen, China have set out to identify the transcription factors that contribute to the immune dysregulation in SLE by determining the chromatin accessibility landscape. By performing single cell analysis they show that 12 transcription factors, regulating 12 immune genes that characterize SLE. The complexity of this disease is illustrated by the fact that different profiles are found in T cells, B cells, monocytes and NK cells.

Glucocorticoids are important endocrine regulators of the immune system and as such used for a number of decades as pharmacological treatment of many autoimmune and autoinflammatory diseases. Ronchetti et al., from the group of Carlo Riccardi in Perugia, Italy, has reviewed the use of glucocorticoids (GC) for rare inflammatory and autoimmune diseases such as insulin autoimmune syndrome, relapsing polychondritis syndrome, dermatomyositis, and hemophagocytic lymphohistiocytosis. Prolonged use of GC comes with severe side-effects and therefore alternative, less toxic drugs would be needed. The potential for GC-induced proteins, such as glucocorticoid-induced leucine zipper (GILZ) for this purpose is discussed.

Relative little attention has been paid to the impact of GC, both physiologically as well as a drug, on regulation of tissue resident macrophages, a topic that is addressed by Diaz-Jimenez et al. from the NIH at Research Triangle Park. In heart tissue, in the central nervous system, in the gastrointestinal tract, and in the liver, glucocorticoids regulate immune surveillance by their effects on tissue resident macrophages. Glucocorticoids also exert their functions *via* GITR, the glucocorticoid-induced TNFR related protein as described by Tian et al. from Zhenjiang, China. As a consequence of GC, GITR is expressed on regulatory T cells and effector T cells as well as on NK cells and neutrophils. GITR is activated when interacting with its ligand, GIRTL, expressed constitutively on B cells and dendritic cells. Abnormalities in GITR/GITRL may contribute to the immune dysregulation in Sjögren’s syndrome. The emerging data indicate that GITR blockade could be a potential treatment option for Sjögren’s syndrome as well as other autoimmune diseases.

TNF plays a central role in many autoimmune and inflammatory diseases, and biological TNF blockade has revolutionized the therapy of rheumatoid arthritis, psoriasis, and Crohn’s disease. In latter disease TNF blockade is effective in 60-70% of patients. Lykowska-Szuber et al. from Poznan University, Poland have studied expression of apoptosis genes in inflamed colon tissue of responder and non-responder Crohn’s disease patients. They find reduced expression of the TNF receptor superfamily member 1B gene, *TNFRSF1B* in refractory patients which explains why these patients do not respond to this therapy.

Maintaining or restoring a balanced immune system is more complicated than keeping a buffered solution at neutral pH. Especially the autoimmune and autoinflammatory diseases are like a poke bowl mixture of genetic, epigenetic, hormonal and environmental ingredients. Detailed knowledge on how, where, and when these ingredients may go wrong will be needed to further our insight into the cellular and molecular mechanisms of immune (dys)regulation. And that will allow for the development and implementation of new immunotherapeutic interventions and further refinement of existing ones.

## Author Contributions

GR, CR, and FK were the guest editors of this Research Topic. GR wrote the first draft. CR and FK commented on and contributed to this editorial. All authors contributed to the article and approved the submitted version.

## Conflict of Interest

The authors declare that the research was conducted in the absence of any commercial or financial relationships that could be construed as a potential conflict of interest.

## Publisher’s Note

All claims expressed in this article are solely those of the authors and do not necessarily represent those of their affiliated organizations, or those of the publisher, the editors and the reviewers. Any product that may be evaluated in this article, or claim that may be made by its manufacturer, is not guaranteed or endorsed by the publisher.

